# Experience-dependent olfactory behaviors of the parasitic nematode *Heligmosomoides polygyrus*

**DOI:** 10.1371/journal.ppat.1006709

**Published:** 2017-11-30

**Authors:** Felicitas Ruiz, Michelle L. Castelletto, Spencer S. Gang, Elissa A. Hallem

**Affiliations:** 1 Department of Microbiology, Immunology, and Molecular Genetics, University of California, Los Angeles, Los Angeles, California, United States of America; 2 Molecular Biology Institute, University of California, Los Angeles, Los Angeles, California, United States of America; University of Glasgow, UNITED KINGDOM

## Abstract

Parasitic nematodes of humans and livestock cause extensive disease and economic loss worldwide. Many parasitic nematodes infect hosts as third-stage larvae, called iL3s. iL3s vary in their infection route: some infect by skin penetration, others by passive ingestion. Skin-penetrating iL3s actively search for hosts using host-emitted olfactory cues, but the extent to which passively ingested iL3s respond to olfactory cues was largely unknown. Here, we examined the olfactory behaviors of the passively ingested murine gastrointestinal parasite *Heligmosomoides polygyrus*. *H*. *polygyrus* iL3s were thought to reside primarily on mouse feces, and infect when mice consume feces containing iL3s. However, iL3s can also adhere to mouse fur and infect orally during grooming. Here, we show that *H*. *polygyrus* iL3s are highly active and show robust attraction to host feces. Despite their attraction to feces, many iL3s migrate off feces to engage in environmental navigation. In addition, *H*. *polygyrus* iL3s are attracted to mammalian skin odorants, suggesting that they migrate toward hosts. The olfactory preferences of *H*. *polygyrus* are flexible: some odorants are repulsive for iL3s maintained on feces but attractive for iL3s maintained off feces. Experience-dependent modulation of olfactory behavior occurs over the course of days and is mediated by environmental carbon dioxide (CO_2_) levels. Similar experience-dependent olfactory plasticity occurs in the passively ingested ruminant-parasitic nematode *Haemonchus contortus*, a major veterinary parasite. Our results suggest that passively ingested iL3s migrate off their original fecal source and actively navigate toward hosts or new host fecal sources using olfactory cues. Olfactory plasticity may be a mechanism that enables iL3s to switch from dispersal behavior to host-seeking behavior. Together, our results demonstrate that passively ingested nematodes do not remain inactive waiting to be swallowed, but rather display complex sensory-driven behaviors to position themselves for host ingestion. Disrupting these behaviors may be a new avenue for preventing infections.

## Author summary

Many parasitic nematodes infect by passive ingestion when the host consumes food, water, or feces containing infective third-stage larvae (iL3s). Passively ingested nematodes that infect humans cause severe gastrointestinal distress and death in endemic regions, and those that infect livestock are a major cause of production loss worldwide. Because these parasites do not actively invade hosts but instead rely on being swallowed by hosts, it has been assumed that they show only limited sensory responses and do not engage in host-seeking behaviors. Here, we investigate the olfactory behaviors of the passively ingested murine parasite *Heligmosomoides polygyrus* and show that this assumption is incorrect; *H*. *polygyrus* iL3s show robust attraction to a diverse array of odorants found in mammalian skin, sweat, and feces. Moreover, the olfactory responses of *H*. *polygyrus* iL3s are experience-dependent: some odorants are repulsive to iL3s cultured on feces but attractive to iL3s removed from feces. Olfactory plasticity is also observed in the ruminant parasite *Haemonchus contortus*, and may enable iL3s to disperse in search of new hosts or host fecal sources. Our results suggest that passively ingested nematodes use olfactory cues to navigate their environments and position themselves where they are likely to be swallowed. By providing new insights into the olfactory behaviors of these parasites, our results may enable the development of new strategies for preventing infections.

## Introduction

Passively ingested gastrointestinal parasitic nematodes of humans and livestock are a significant health and economic problem. Human-infective nodular worms in the genus *Oesophagostomum* are a growing health concern in endemic regions of Africa, where they can cause abdominal pain, weight loss, diarrhea, and death [1–3]. Passively ingested parasites of livestock result in decreased production and economic loss worldwide. For example, *Haemonchus contortus* is an important parasite of ruminants that causes gastrointestinal distress, anemia, edema, and death in livestock [4]. In the United States alone, over 2.7 million goats and 2.6 million sheep are infected with *H*. *contortus* [5]. Infections with these parasites can be cleared using anthelmintic drugs, but frequent administration has led to increased drug resistance [6–9]. Although the host immune response to infection with passively ingested nematodes is well-studied [10–12], remarkably little is known about the behaviors of the parasites themselves. A better understanding of the behaviors exhibited by the environmental life stages of these parasites could facilitate the development of new strategies for preventing infections of humans and livestock, such as the use of targeted traps or repellents.

Parasitic nematodes that actively invade hosts by skin penetration are known to engage in sensory-driven host seeking [13]. For example, the human hookworms *Ancylostoma duodenale* and *Necator americanus*, and the dog hookworm *Ancylostoma caninum*, are relatively inactive in the absence of sensory stimuli but show increased activity in the presence of heat, CO_2_, and/or skin extract [14–16]. Hookworms also migrate robustly toward a heat source [14, 17]. The human, non-human primate, and canine threadworm *Strongyloides stercoralis*, and the rat parasites *Strongyloides ratti* and *Nippostrongylus brasiliensis*, also respond robustly to host-emitted sensory cues. They are active in the absence of sensory stimuli [18], and show robust attraction to a wide variety of odorants emitted by human skin and sweat [18–20]. *S*. *ratti* is also known to be attracted to blood serum, and *S*. *stercoralis* to blood serum, sweat, and heat [19, 21, 22].

The sensory behaviors of passively ingested nematodes are much less understood. Some passively ingested worms are capable of responding to environmental sensory cues such as temperature, humidity, and odorants [13]. For example, *H*. *contortus* uses temperature and humidity cues to migrate vertically through grass in response to changes in environmental conditions [23, 24]. Because passively ingested worms do not actively invade hosts, it has often been assumed that they do not host seek and do not respond to host-emitted sensory cues. However, we recently showed that *H*. *contortus* is attracted to some host-emitted odorants, raising the possibility that it can use olfactory cues to position itself in the vicinity of potential hosts [18]. Since many hosts develop immunity to passively ingested worms following repeated infection [25, 26], behaviors that expose these parasites to new hosts may be important for parasite propagation.

Here, we use the passively ingested gastrointestinal murine parasite *H*. *polygyrus* (also called *H*. *bakeri* [27, 28]) as a model system for studying the sensory behaviors of passively ingested gastrointestinal nematodes, and for testing the hypothesis that passively ingested nematodes engage in host seeking. As a mouse parasite, *H*. *polygyrus* is one of the only passively ingested nematodes that can be easily maintained in the lab [29, 30]. *H*. *polygyrus* is only infective as developmentally arrested iL3s, which are analogous to *Caenorhabditis elegans* dauers (S1 Fig) [31]. *H*. *polygyrus* iL3s were thought to primarily reside in host feces and infect when mice, which are coprophagic, eat infested feces [32, 33]. However, *H*. *polygyrus* iL3s can also attach to mouse fur and be ingested during grooming [34]. *H*. *polygyrus* iL3s were previously shown to nictate [33, 34], a behavior where the iL3 stands on its tail and waves its head [13], which may increase the probability of being swallowed during coprophagy or of becoming attached to mouse fur [34]. Once inside the host, the nematodes grow to adulthood and reproduce in the host intestine. *H*. *polygyrus* eggs then exit the host in feces and develop there into iL3s capable of infecting new hosts.

The fact that *H*. *polygyrus* develops on feces and infects mice from feces raises the question of whether *H*. *polygyrus* iL3s engage in environmental navigation using either host-emitted or environmental sensory cues, or whether they simply remain on feces and wait to be ingested. While this question had not been investigated thoroughly, *H*. *polygyrus* iL3s were previously found to be attracted to mouse urine and skin lipids, suggesting they are capable of responding to at least some host sensory cues [34]. However, the extent to which *H*. *polygyrus* iL3s engage in sensory behaviors that increase the likelihood that they will be swallowed by hosts remained unclear.

To address this question, we conducted a large-scale quantitative analysis of the unstimulated and odor-stimulated behaviors of *H*. *polygyrus*. We found that *H*. *polygyrus* iL3s were active in the absence of odor stimulation. In addition, they were attracted to host fecal odor. While they showed robust attraction to fresh feces, they showed reduced attraction to aged feces and ultimately migrated off their original fecal source to engage in environmental navigation. *H*. *polygyrus* iL3s were attracted to skin odorants as well as fecal odorants, suggesting that they are capable of migrating toward hosts as well as new host fecal sources. In addition, *H*. *polygyrus* iL3s showed experience-dependent olfactory plasticity, such that some host-emitted odorants were repulsive to iL3s cultured on feces but attractive to iL3s cultured off feces. Olfactory plasticity was also observed in the ruminant parasite *H*. *contortus*, and may be a general mechanism that enables passively ingested iL3s to shift from dispersal behavior to host-seeking behavior. Our results suggest that passively ingested nematodes disperse from feces and engage in host seeking to position themselves where they are likely to be ingested by new hosts.

## Results

### *H*. *polygyrus* iL3s are active in the absence of sensory stimulation

**Fig 1:**
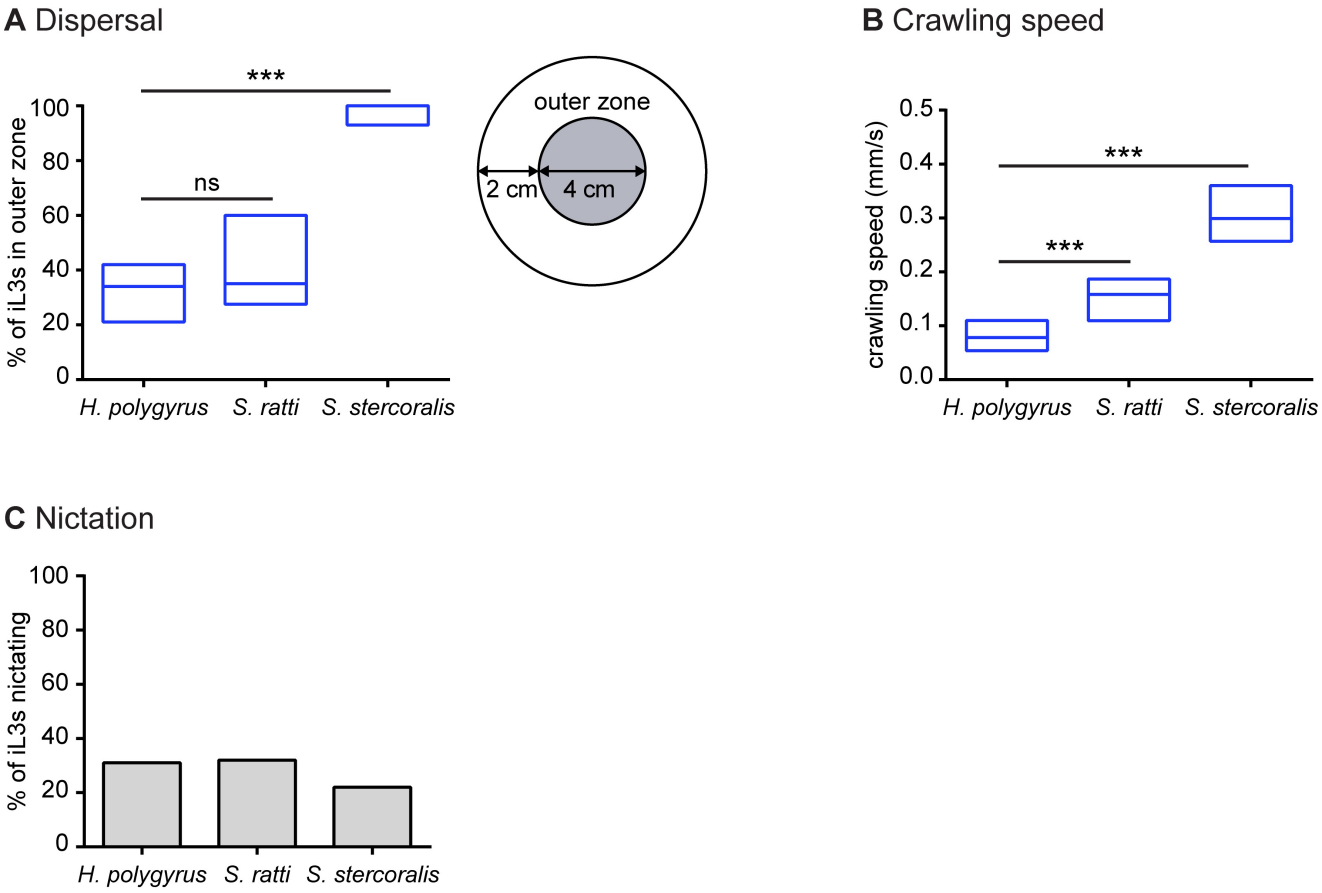
Navigational strategies of *H*. *polygyrus* in comparison to those of *S*. *ratti* and *S*. *stercoralis*. **A**. Dispersal behavior across species. iL3s were placed at the center of an agar plate and allowed to crawl freely for 1 hour in the absence of applied sensory stimuli. The percentage of iL3s in the outer zone, defined as the region of the plate outside a 4-cm-diameter circle (right), was determined. *H*. *polygyrus* and *S*. *ratti* iL3s dispersed to a similar extent, while *S*. *stercoralis* iL3s dispersed to a greater extent. ***p<0.001, Kruskal-Wallis test with Dunn’s post-test. n = 9–11 trials for each species and condition. **B**. Crawling speed across species. *H*. *polygyrus* iL3s crawled more slowly than *S*. *ratti* and *S*. *stercoralis* iL3s. ***p<0.001, one-way ANOVA with Holm-Sidak’s post-test. n = 23–31 iL3s per species. For **A**-**B**, graphs show medians and interquartile ranges. **C**. Nictation frequencies were similar across species (*p* = 0.65, chi-square test). n = 22–70 iL3s per species. Data for *S*. *ratti* and *S*. *stercoralis* are from Castelletto *et al*., 2014 [18].

Parasitic nematodes are known to vary in their environmental navigation strategies: some are cruisers that actively navigate toward hosts; some are ambushers that are less active and primarily attach to passing hosts; and some use an intermediate strategy [13]. To gain insight into the movement strategy used by *H*. *polygyrus*, we first examined the unstimulated movement of *H*. *polygyrus* iL3s, and compared their movement to that of *S*. *stercoralis* and *S*. *ratti* iL3s, which are known to be cruisers [18]. Using a dispersal assay in which iL3s were allowed to migrate on an agar surface in the absence of applied sensory stimulation for 1 hour, we found that *H*. *polygyrus* iL3s and *S*. *ratti* iL3s dispersed to a similar extent, whereas *S*. *stercoralis* iL3s dispersed more than either rodent parasite (Fig 1A). These results demonstrate that *H*. *polygyrus* iL3s are active in the absence of sensory stimulation and are capable of exhibiting a movement strategy resembling that of a cruiser. The increased movement of *S*. *stercoralis* iL3s relative to *H*. *polygyrus* and *S*. *ratti* iL3s may reflect the larger habitats of humans relative to nesting rodents [18]; since nesting rodents spend more time near their fecal deposits than do humans, non-human primates, and dogs, *S*. *stercoralis* iL3s may need to disperse farther into the environment to successfully locate a host.

Dispersal behavior reflects both crawling speed and other parameters such as crawling trajectory and tendency to pause during crawling. To gain more insight into the navigational strategy used by *H*. *polygyrus* iL3s, we tracked their crawling speed using automated worm tracking [35]. We found that *H*. *polygyrus* iL3s crawled more slowly than *S*. *ratti* iL3s, while *S*. *stercoralis* iL3s crawled much more rapidly than the rodent parasites (Fig 1B). The ability of *H*. *polygyrus* iL3s to disperse to the same extent as *S*. *ratti* iL3s despite their slower crawling speed suggests that *H*. *polygyrus* iL3s exhibit more linear and/or continuous movement than *S*. *ratti* iL3s.

We also evaluated the nictation behavior of *H*. *polygyrus*. Many skin-penetrating and passively ingested iL3s engage in nictation, a common ambushing behavior, as a means of increasing host contact. By standing up on a surface, nictating iL3s are more likely to touch and then transfer onto a passing host, or to be swallowed by a foraging host [13]. We assayed the nictation behavior of *H*. *polygyrus*, and compared it to that of *S*. *ratti* and *S*. *stercoralis*, using “micro-dirt” agar chips with near-microscopic pillars as an artificial dirt substrate (S2 Fig) [36]. The pillars on the agar surface minimize surface tension, allowing the iL3s to stand. We found that all three of the species showed similarly low nictation frequencies: only ~20–30% of the tested iL3s nictated during the assay period (Fig 1C). The low nictation frequencies of *S*. *ratti* and *S*. *stercoralis* are consistent with a cruising navigational strategy [18]. The similarly low nictation frequency of *H*. *polygyrus*, combined with its active crawling behavior, suggests that it also behaves more like a cruiser than an ambusher. These results demonstrate that passively ingested iL3s do not remain inactive waiting to be swallowed by passing hosts. Rather, like skin-penetrating iL3s, they engage in environmental navigation.

### *H*. *polygyrus* iL3s are attracted to host feces

**Fig 2:**
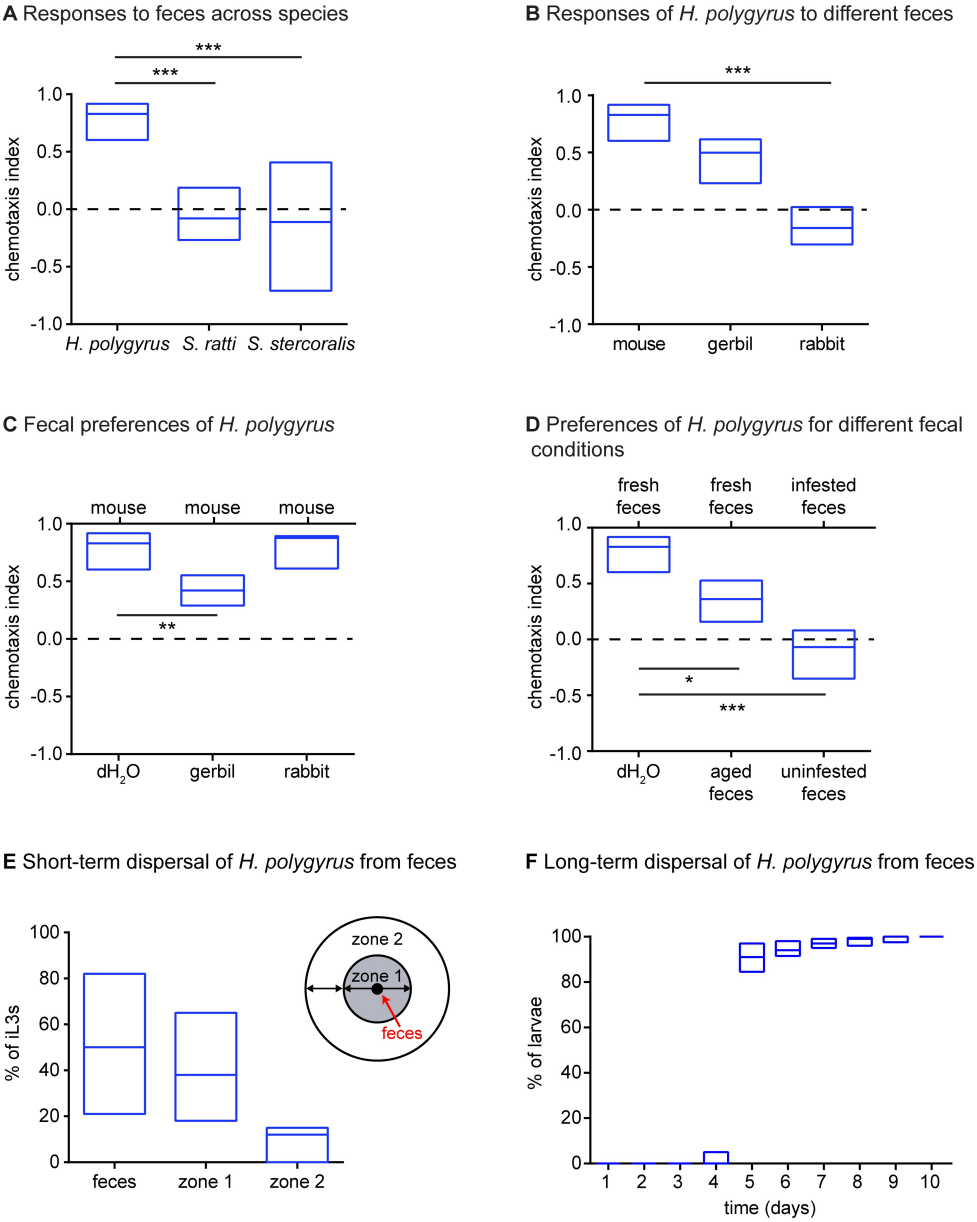
*H*. *polygyrus* iL3s are attracted to host feces. **A**. *H*. *polygyrus* iL3s were attracted to mouse feces. By contrast, *S*. *ratti* and *S*. *stercoralis* iL3s were not attracted to the feces of their hosts (rat and dog, respectively). ***p<0.001, Kruskal-Wallis test with Dunn’s post-test. n = 12–14 trials for each species. Data for *S*. *ratti* and *S*. *stercoralis* are from Castelletto *et al*., 2014 [18]. **B-C**. *H*. *polygyrus* iL3s respond differently to feces from different animals (**B**), and prefer mouse feces to gerbil or rabbit feces (**C**). Labels above and below each box in **C** indicate the opposing cues in the fecal preference assay. **p<0.01, ***p<0.001, Kruskal-Wallis test with Dunn’s post-test. n = 10–14 trials per condition. **D**. *H*. *polygyrus* iL3s prefer fresh mouse feces to aged mouse feces, but cannot distinguish mouse feces from infected animals versus uninfected animals. *p<0.05, ***p<0.001, Kruskal-Wallis test with Dunn’s post-test. n = 11–14 trials per condition. **E**. In a short-term dispersal assay, *H*. *polygyrus* iL3s leave feces to engage in host seeking. iL3s were placed on fresh mouse feces and allowed to crawl freely for 1 hour. The number of iL3s either on the feces, in zone 1, or in zone 2 (right) was then quantified. Approximately half of the iL3 population migrated off of the feces. n = 11 trials, with 15–40 iL3s per trial. **F**. In a long-term dispersal assay, nearly all *H*. *polygyrus* iL3s eventually left their original fecal pellet to engage in host seeking. The cumulative percentage of nematodes that had migrated off of their original fecal pellet was quantified each day over the course of 10 days. n = 13 trials. For all graphs, lines indicate medians and interquartile ranges.

If passively ingested iL3s utilize active strategies to position themselves in optimal locations for host ingestion, one strong prediction is that the species that infect coprophagic hosts (*e*.*g*., mice) will be attracted to host feces. We examined the response of *H*. *polygyrus* iL3s to fresh fecal odor using a chemotaxis assay in which the iL3s could smell but not make contact with the feces. We found that *H*. *polygyrus* iL3s were strongly attracted to fresh mouse feces (Fig 2A and 2B). Moreover, they preferred mouse feces to gerbil or rabbit feces (Fig 2B and 2C), indicating that they can distinguish host from non-host feces. By contrast, *S*. *stercoralis* and *S*. *ratti* iL3s were neutral to host feces (Fig 2A) [18]. The different responses of *H*. *polygyrus* and *Strongyloides* iL3s to fecal odor are understandable in the context of their different lifestyles. Although the pre-infective larvae of both *H*. *polygyrus* and *Strongyloides* inhabit host feces, *H*. *polygyrus* iL3s can infect hosts from feces while skin-penetrating iL3s must migrate off feces and onto host skin [13, 30]. Thus, attraction to host feces would likely be ecologically advantageous for *H*. *polygyrus* iL3s but not *Strongyloides* iL3s. In addition, we found that *H*. *polygyrus* iL3 were more attracted to fresh feces than aged feces (Fig 2D), suggesting that the iL3s use olfaction to identify favorable fecal sources. In contrast, they did not show a preference for feces from uninfected versus infected hosts (Fig 2D), suggesting that they are attracted to fresh host feces regardless of the infection status of the host. Attraction of *H*. *polygyrus* iL3s to fecal odor may cause some of the iL3s on a fresh fecal source to remain there, and may draw iL3s from fecal sources that have become suboptimal due to age, desiccation, or other conditions.

### *H*. *polygyrus* iL3s disperse from feces to engage in environmental navigation

The robust attraction of *H*. *polygyrus* iL3s to fecal odor raised the question of whether the iL3s leave feces under normal conditions. To address this question, we performed two different fecal dispersal assays, the first to assess short-term dispersal over the course of hours and the second to assess long-term dispersal over the course of days. In the short-term dispersal assay, iL3s were placed on fresh feces in the center of an agar surface. The frequency with which the iL3s migrated off the feces and onto the agar was then quantified. We found that on average, 50% of the iL3 population left the fresh feces; in some trials, over 80% of the iL3s left the feces (Fig 2E). These results demonstrate that even for iL3s on fresh feces, which are presumably a favorable fecal source, a substantial portion of the iL3 population migrates off of the feces and engages in environmental navigation.

In the long-term dispersal assay, a fresh fecal pellet from an infected animal was collected, and one-half of the pellet was placed in the center of an agar surface. The frequency with which the nematodes migrated off of the feces and onto the agar was then quantified each day for a period of 10 days. Thus, this assay examined *H*. *polygyrus* dispersal in the more natural context of fecal aging. We found that nearly all of the nematodes remained on the feces until day 5. On day 5, by which time the nematodes had developed into iL3s [29], over 80% of the nematodes migrated off the feces (Fig 2F). By day 10, nearly 100% of the nematodes had migrated off of the feces (Fig 2F). In the same assay, we also examined nictation behavior and found that nictation occurs primarily on day 5 (S3 Fig), at the time when the majority of the population migrates off of the feces (Fig 2F). Together, these results argue against the possibility that some members of the iL3 population are ambushers while others are cruisers, and suggest instead that nearly all *H*. *polygyrus* iL3s ultimately engage in cruising behavior.

### *H*. *polygyrus* iL3s are attracted to mammalian-derived odorants

**Fig 3:**
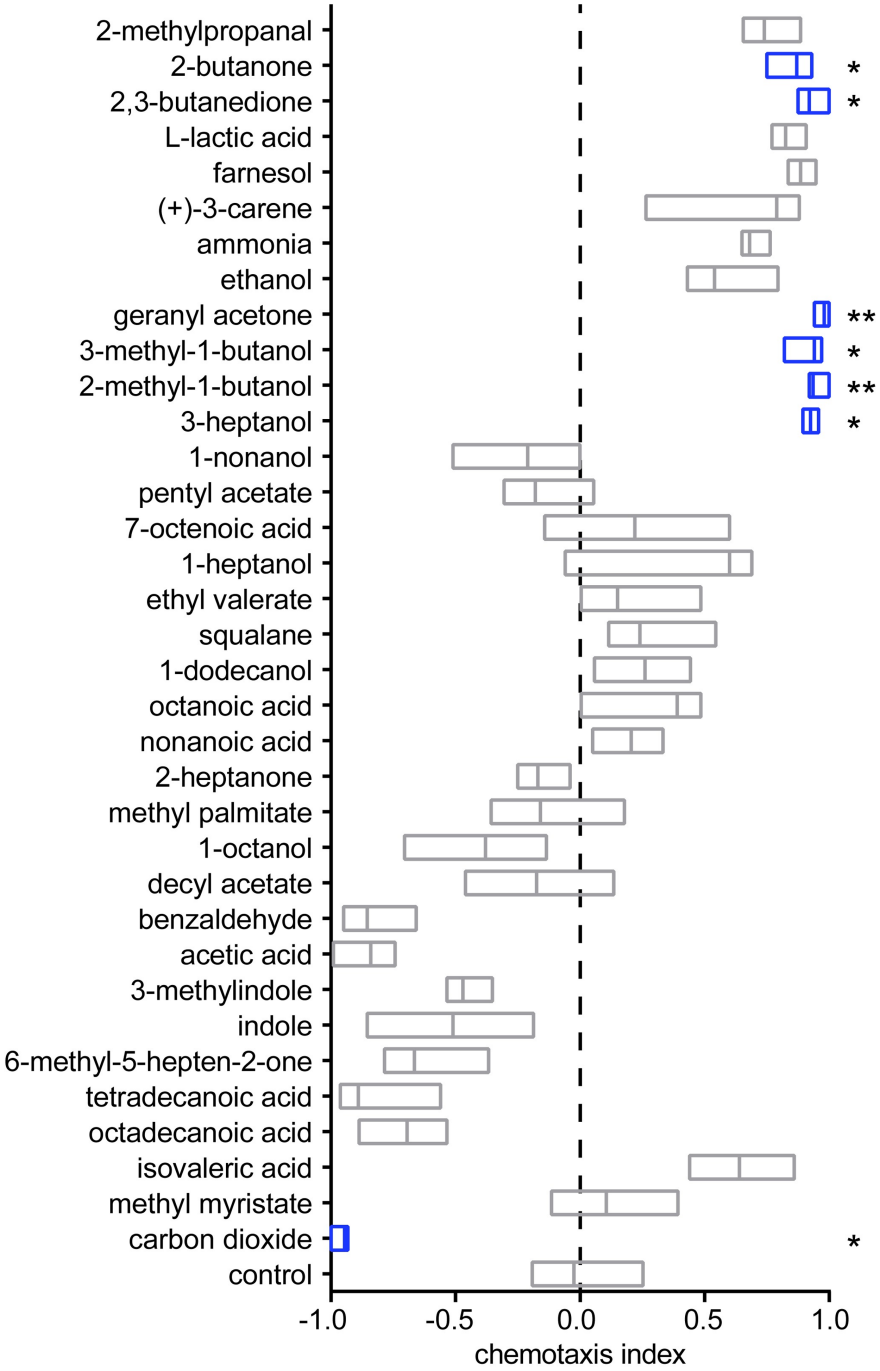
*H*. *polygyrus* iL3s are attracted to mammalian odorants. *H*. *polygyrus* iL3s were attracted to 6 of 35 tested odorants and were repelled by CO_2_ in a chemotaxis assay. *p<0.05 and **p<0.01 relative to the paraffin oil control, Kruskal-Wallis test with Dunn’s post-test. Significant responses are highlighted in blue. n = 8–28 trials for each condition. Lines indicate medians and interquartile ranges.

Our results show that *H*. *polygyrus* iL3s will eventually leave their original fecal source and migrate toward new fecal sources to position themselves for ingestion during coprophagy. However, *H*. *polygyrus* iL3s can infect during grooming [34], raising the question of whether they also migrate toward hosts by detecting host-emitted olfactory cues. To investigate this possibility, we examined the responses of *H*. *polygyrus* iL3s to a large panel of odorants that included compounds found in mammalian skin and sweat using a chemotaxis assay (S4 Fig) [18]. We found that *H*. *polygyrus* iL3s showed robust attraction to 6 of the 35 odorants tested: 2-butanone; 2,3-butanedione; geranyl acetone; 3-methyl-1-butanol; 2-methyl-1-butanol; and 3-heptanol (Fig 3). In contrast, CO_2_ was repulsive for *H*. *polygyrus* iL3s (Fig 3). All of the attractive odorants are emitted from mammalian skin, feces, and/or urine [18, 37–41]. Notably, 2-methyl-1-butanol, 3-methyl-1-butanol, and geranyl acetone are present in skin microbiota [42, 43] and are known attractants for skin-penetrating nematodes [18]. Attraction to these odorants could drive migration of *H*. *polygyrus* iL3s toward hosts.

To gain insight into how the olfactory preferences of *H*. *polygyrus* iL3s differ from those of other iL3s that engage in environmental navigation, we compared the odor-driven behaviors of *H*. *polygyrus* to those of 7 other nematode species: the skin-penetrating human-parasitic nematode *S*. *stercoralis*, the skin-penetrating rat-parasitic nematodes *S*. *ratti* and *N*. *brasiliensis*, the passively ingested ruminant-parasitic nematode *H*. *contortus*, the actively invading entomopathogenic nematodes *Heterorhabditis bacteriophora* and *Steinernema carpocapsae*, and the free-living bacterivorous nematode *C*. *elegans*. This comparison revealed that *H*. *polygyrus* responds differently to the odorant panel than the other species (S5A Fig), consistent with previous studies demonstrating that parasitic nematodes show species-specific olfactory preferences [18, 44, 45]. Moreover, cluster analysis of the 8 species based on their olfactory preferences revealed that parasitic nematodes that infect the same hosts have more similar olfactory preferences than parasitic nematodes that infect different hosts (S5B Fig) [18, 44, 45]. In contrast, parasitic nematodes that infect different hosts but share the same mode of infection do not respond similarly to odorants. In particular, *H*. *polygyrus* and *H*. *contortus* are both passively ingested but infect different hosts, and their olfactory preferences are dissimilar (S5B Fig). Thus, olfactory preferences appear to be determined primarily by host range rather than infection mode. The fact that distantly related species that target the same host respond similarly to odorants strongly suggests that parasitic nematode olfactory behavior has evolved to mediate specific parasite-host interactions.

### The navigational strategies of *H*. *polygyrus* iL3s are shaped by their recently experienced environment

**Fig 4:**
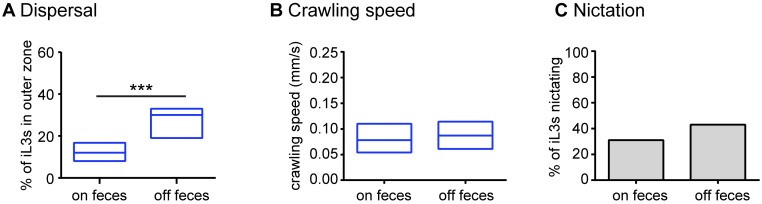
The navigational strategies of *H*. *polygyrus* are experience-dependent. **A**. Dispersal behavior of iL3s cultured on vs. off feces. *H*. *polygyrus* iL3s cultured off feces dispersed to a greater extent than *H*. *polygyrus* iL3s cultured on feces. ***p<0.001, Mann-Whitney test. n = 17–36 trials per condition. Dispersal was assayed after 10 minutes. The outer zone is as defined in Fig 1A. **B**. Crawling speed did not differ for iL3s cultured on vs. off feces (*p* = 0.82, Mann-Whitney test). n = 23–31 iL3s per condition. For A-B, graphs show medians and interquartile ranges. **C**. Nictation frequency did not differ for iL3s cultured on vs. off feces (*p* = 0.21, Fisher’s exact test). Graph shows the percentage of animals that nictated for each condition. Data were analyzed as a contingency table. n = 61–70 iL3s for each condition. For **A**-**C**, nematodes were allowed to develop on feces for 7 days until they reached the iL3 stage. The “on-feces” iL3s were then cultured on feces for 7 additional days, while the “off-feces” iL3s were cultured in dH_2_O for 7 days. iL3s were tested on day 14.

iL3s that have migrated off feces likely face a greater ethological drive to search for new hosts or fecal sources than iL3s that have remained on feces. We therefore wondered whether iL3s that have migrated off feces might exhibit different behaviors than iL3s on feces. To test this possibility, we compared the unstimulated migration of iL3s cultivated on feces to those of iL3s that had been removed from feces and maintained in dH_2_O for 1 week. We found that the off-feces iL3s dispersed to a greater extent than the on-feces iL3s (Fig 4A), demonstrating that the unstimulated activity of *H*. *polygyrus* iL3s is subject to experience-dependent modulation. The greater dispersal of off-feces iL3s was not due to changes in crawling speed (Fig 4B); thus, the difference in dispersal reflects a difference in navigational strategy rather than motility. In addition, the nictation rate of on-feces vs. off-feces iL3s was unchanged (Fig 4C), demonstrating that removal from feces results in a specific change in crawling behavior. The increased dispersal of off-feces iL3s likely increases the probability of encountering a new host or fecal source.

### *H*. *polygyrus* iL3s show experience-dependent olfactory plasticity

**Fig 5:**
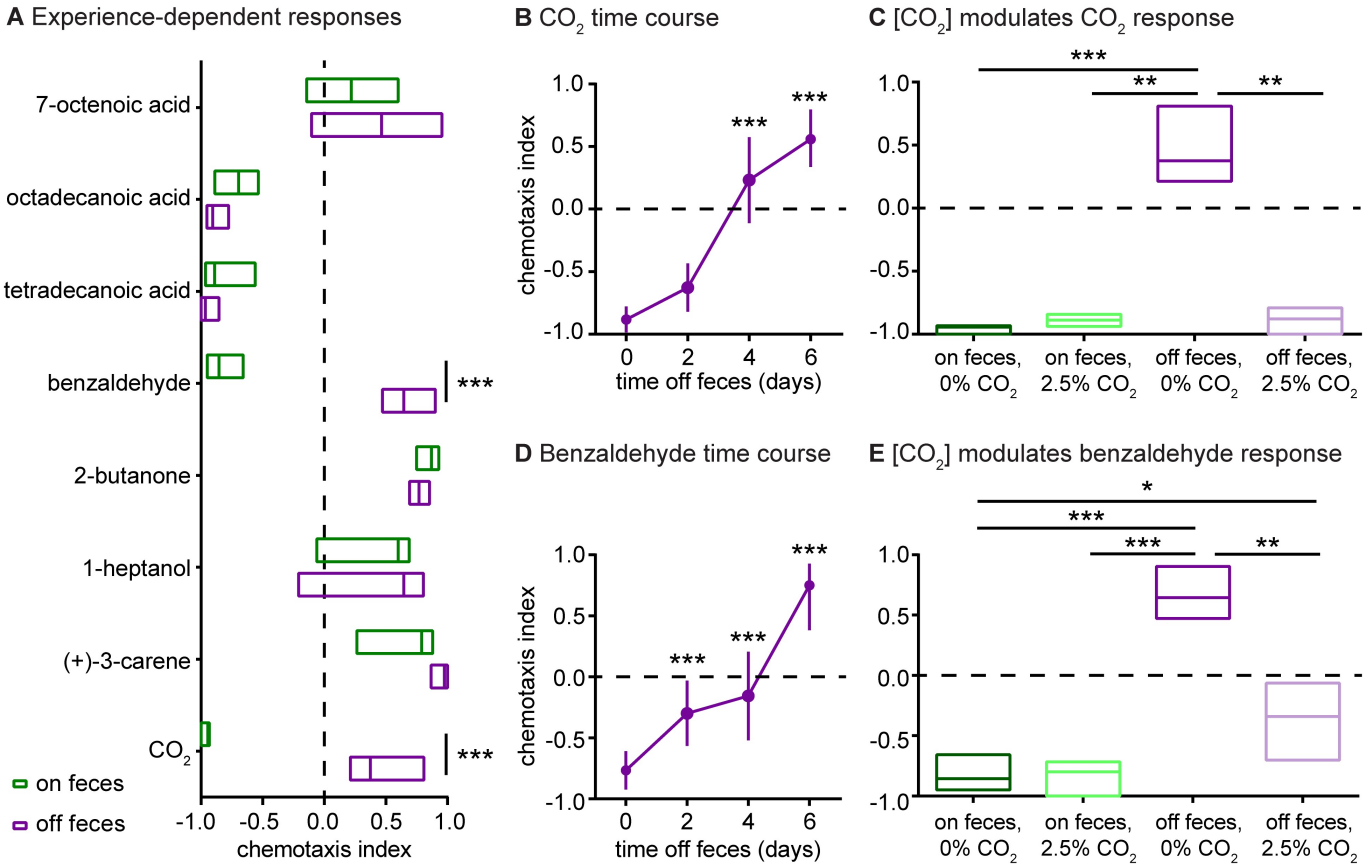
*H*. *polygyrus* iL3s exhibit experience-dependent olfactory plasticity. **A**. iL3s cultured on vs. off feces respond differently to a subset of odorants in a chemotaxis assay. Benzaldehyde and CO_2_ were repulsive for on-feces iL3s but attractive for off-feces iL3s. ***p<0.001, two-way ANOVA with Sidak’s post-test. n = 8–28 trials for each condition. **B**. CO_2_-response valence shifted from repulsive to attractive over the course of 6 days following removal from feces. Day 0 indicates the time of removal from feces. ***p<0.001 relative to day 0, Kruskal-Wallis test with Dunn’s post-test. n = 12–18 trials for each condition. **C**. Environmental CO_2_ levels determine CO_2_-response valence. iL3s cultured on feces at either ambient CO_2_ (“0% CO_2_”) or high CO_2_ (“2.5% CO_2_”) were repelled by 10% CO_2_, iL3s cultured off feces at 0% CO_2_ were attracted to 10% CO_2_, and iL3s cultured off feces at 2.5% CO_2_ were repelled by 10% CO_2_. **p<0.01, ***p<0.001, Kruskal-Wallis test with Dunn’s post-test. n = 10–12 trials for each condition. **D**. Benzaldehyde-response valence shifted from repulsive to attractive over the course of 6 days following removal from feces. ***p<0.001 relative to day 0, one-way ANOVA with Dunnett’s post-test. n = 10–16 trials for each condition. **E**. Environmental CO_2_ levels determine benzaldehyde-response valence. iL3s cultured on feces at either 0% CO_2_ or 2.5% CO_2_ were repelled by benzaldehyde, iL3s cultured off feces at 0% CO_2_ were attracted to benzaldehyde, and iL3s cultured off feces at 2.5% CO_2_ were slightly repelled by benzaldehyde. *p<0.05, **p<0.01, ***p<0.001, Kruskal-Wallis test with Dunn’s post-test. n = 10–28 trials for each condition. Graphs show medians and interquartile ranges.

To further elucidate the effects of recently experienced environment on *H*. *polygyrus* behavior, we compared the olfactory preferences of on-feces vs. off-feces iL3s to a subset of mammalian odorants. The odorant panel was selected to include attractive, neutral, and repulsive odorants. We found that on-feces and off-feces iL3s responded differently to 2 of 8 tested odorants: CO_2_ and benzaldehyde. Both odorants were repulsive for iL3s on feces but attractive for iL3s off feces (Fig 5A). For both on-feces and off-feces iL3s, CO_2_-response valence, *i*.*e*. whether CO_2_ was repulsive or attractive, was consistent across concentrations (S6 Fig). CO_2_ is a critical host cue for many parasites, including many parasitic nematodes [13]; it is present at high concentrations in both exhaled breath and feces. Benzaldehyde is found in skin, breath, urine, and feces [18]. Thus, the olfactory responses of *H*. *polygyrus* iL3s to some host-associated odorants are subject to experience-dependent modulation as a result of recently experienced environmental conditions.

We then examined the relationship between cultivation environment and sensory behavior in more detail by investigating the time course of the change in CO_2_- and benzaldehyde-response valence. We found that CO_2_-response valence changed gradually over the course of days when iL3s were removed from feces (Fig 5B). Moreover, culturing iL3s under high CO_2_ conditions prevented the shift in CO_2_-response valence following removal from feces. While iL3s cultured off feces at ambient CO_2_ (~0.04% CO_2_ [46]) were attracted to CO_2_, iL3s cultured off feces at high CO_2_ (2.5% CO_2_) were repelled by CO_2_ (Fig 5C). Thus, CO_2_-response valence is regulated by environmental CO_2_ levels. Benzaldehyde-response valence also changed gradually over the course of days upon removal from feces and was also determined by environmental CO_2_ levels (Fig 5D and 5E). These results suggest that the level of environmental CO_2_ acts as a general regulator of olfactory behavior. Given that feces emit high levels of CO_2_ [39], *H*. *polygyrus* iL3s may use environmental CO_2_ levels to signal the presence or absence of feces, with the result that exposure to high CO_2_ levels mimics the effects of exposure to feces. Experience-dependent olfactory plasticity may be a mechanism that enables iL3s on feces to disperse from the feces, and iL3s that have been off feces for a prolonged period to instead migrate toward new hosts or fresh fecal sources.

### *H*. *contortus* iL3s also show experience-dependent olfactory plasticity

**Fig 6:**
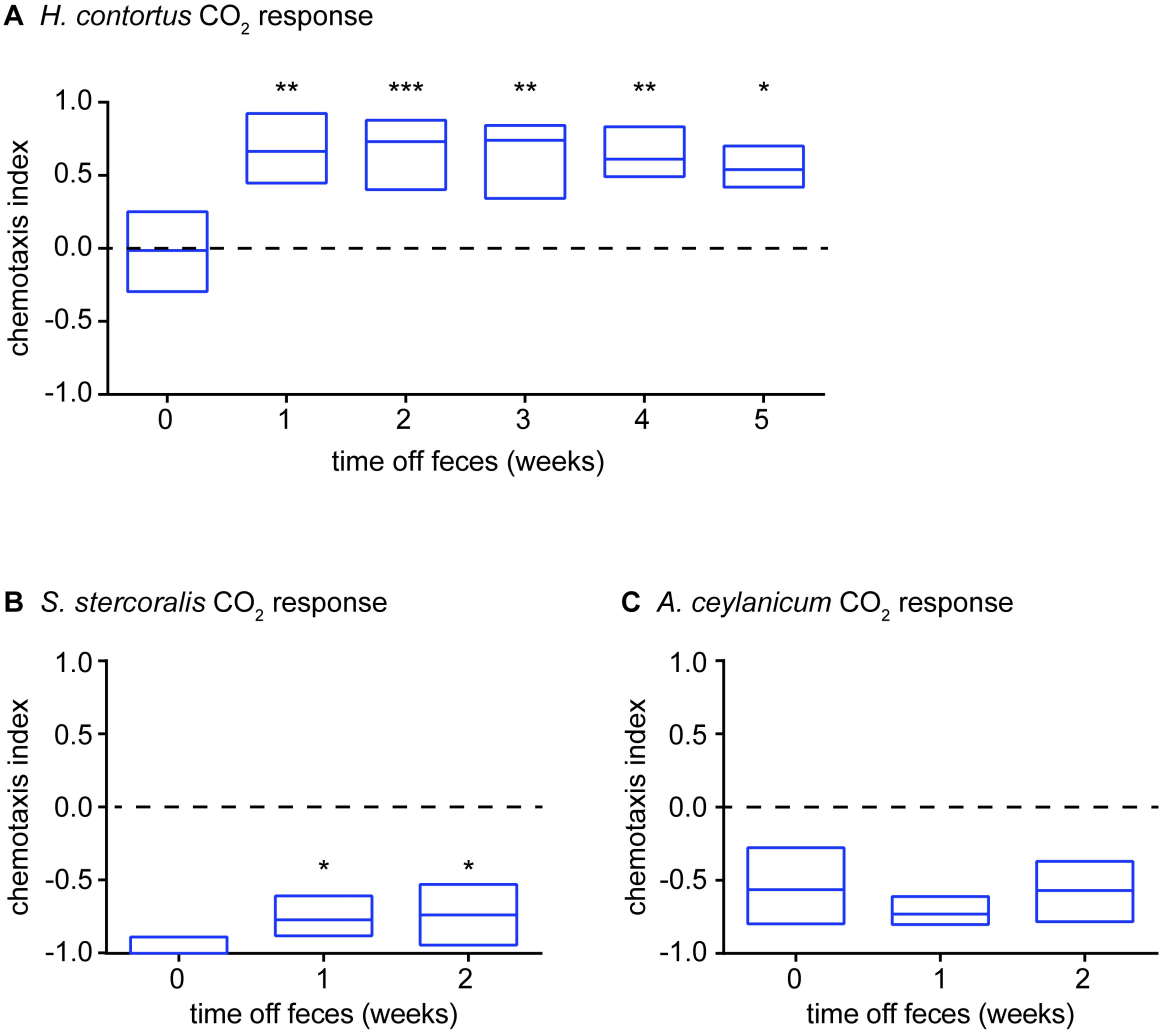
Passively ingested nematodes but not skin-penetrating nematodes show experience-dependent modulation of CO_2_-response valence. **A**. Passively ingested *H*. *contortus* iL3s are neutral to CO_2_ when cultured on feces, but attracted to CO_2_ when cultured off feces in dH_2_O. CO_2_ attraction occurs within 1 week following removal from feces, and is maintained for at least 5 weeks. Nematodes were allowed to develop on feces for at least 7 days. They were then either tested immediately, or stored in dH_2_O for up to 5 weeks and then tested. The “on-feces” group included iL3s aged up to 9 weeks, confirming that CO_2_ attraction occurred as a result of removal from feces rather than increased iL3 age. *p<0.05, **p<0.01, ***p<0.001, Kruskal-Wallis test with Dunn’s post-test. n = 8–16 trials for each condition. **B-C**. Skin-penetrating *S*. *stercoralis* (**B**) and *A*. *ceylanicum* (**C**) iL3s do not show experience-dependent modulation of CO_2_-response valence. Nematodes were allowed to develop on feces for 7 days (**B**) or 10 days (**C**) until they reached the iL3 stage. iL3s were then either tested immediately, stored in BU saline [64] for 7 days and then tested, or stored in BU saline for 14 days and then tested. CO_2_ response was slightly attenuated in *S*. *stercoralis* iL3s cultured off feces, but did not shift from repulsion to attraction (**B**); CO_2_ response was unchanged in *A*. *ceylanicum* iL3s cultured on vs. off feces (**C**). *p<0.05, Kruskal-Wallis test with Dunn’s post-test (**B**) or one-way ANOVA with Dunnett’s post-test (**C**). n = 8–10 trials for each condition. Graphs show medians and interquartile ranges.

Our finding that *H*. *polygyrus* iL3s exhibit experience-dependent olfactory plasticity raised the question of whether this behavior is unique to *H*. *polygyrus* or shared with other parasitic nematode species. To distinguish between these possibilities, we examined the CO_2_-evoked behaviors of *H*. *contortus*, *S*. *stercoralis*, and the skin-penetrating human-parasitic hookworm *Ancylostoma ceylanicum* cultured on versus off feces. We found that like *H*. *polygyrus* iL3s, *H*. *contortus* iL3s show experience-dependent plasticity in their response to CO_2_. In the case of *H*. *contortus*, CO_2_ is neutral for iL3s cultured on feces but attractive for iL3s cultured off feces (Fig 6A). Since *H*. *contortus* iL3s are long-lived [47, 48], sometimes surviving in the environment for up to 8 months [48], we examined the CO_2_-evoked behavior of off-feces iL3s over the course of 5 weeks. We found that CO_2_ changed from neutral to attractive after 1 week, and then remained attractive in subsequent weeks (Fig 6A). Thus, CO_2_ remains a strong attractant for *H*. *contortus* iL3s that have been removed from feces for prolonged periods. Our results demonstrate that experience-dependent olfactory plasticity is not unique to *H*. *polygyrus*, but also occurs in other passively ingested nematodes. Experience-dependent modulation of CO_2_ response may enable *H*. *contortus* iL3s to first migrate off feces and then navigate toward grazing hosts, which emit high concentrations of CO_2_ in their exhaled breath.

In contrast to the passively ingested nematodes, the skin-penetrating nematodes tested did not show experience-dependent modulation of their CO_2_-evoked behavior. Both *S*. *stercoralis* iL3s and *A*. *ceylanicum* iL3s were repelled by CO_2_ when cultured both on and off feces (Fig 6B and 6C). The lack of flexibility in their CO_2_-evoked behavior may reflect the fact that CO_2_ attraction would likely not facilitate host finding by skin-penetrating worms, since very low levels of CO_2_ are given off by the skin [49]. CO_2_ avoidance may function as a dispersal mechanism to drive skin-penetrating iL3s off host feces; attraction to other sensory cues, such as skin and sweat odorants, may then drive the iL3s toward potential hosts [13]. Thus, the ability to exhibit flexible responses to CO_2_ may be a specific behavioral adaptation of passively ingested but not skin-penetrating nematodes.

## Discussion

Here we conducted the first large-scale quantitative behavioral analysis of *H*. *polygyrus* iL3s. We found that *H*. *polygyrus* iL3s were active even in the absence of sensory stimulation (Fig 1). These results argue against the classical notion that passively ingested iL3s remain stationary and wait to be swallowed, and suggest instead that these iL3s actively navigate their environments. We previously showed that *H*. *contortus* iL3s are less active than *S*. *ratti* and *S*. *stercoralis* iL3s [18]. However, the similar dispersal behaviors and nictation rates of *H*. *polygyrus* and *S*. *ratti* (Fig 1) suggest that some passively ingested nematodes are as active as skin-penetrating nematodes despite their passive mode of infection.

Our examination of the olfactory preferences of *H*. *polygyrus* iL3s revealed that they are attracted to fecal odor as well as mammalian skin and sweat odorants (Figs 2 and 3). These results suggest that passively ingested iL3s engage in odor-driven host seeking to position themselves near hosts or host feces, where they are likely to be ingested. Consistent with the attraction of *H*. *polygyrus* iL3s to both fecal odor and host odorants, *H*. *polygyrus* iL3s have been shown to infect hosts either from feces during coprophagy or from fur during grooming [30, 32–34]. Thus, active migration toward new hosts or fecal sources may be a critical but often overlooked aspect of the environmental stage of the *H*. *polygyrus* life cycle.

**Fig 7:**
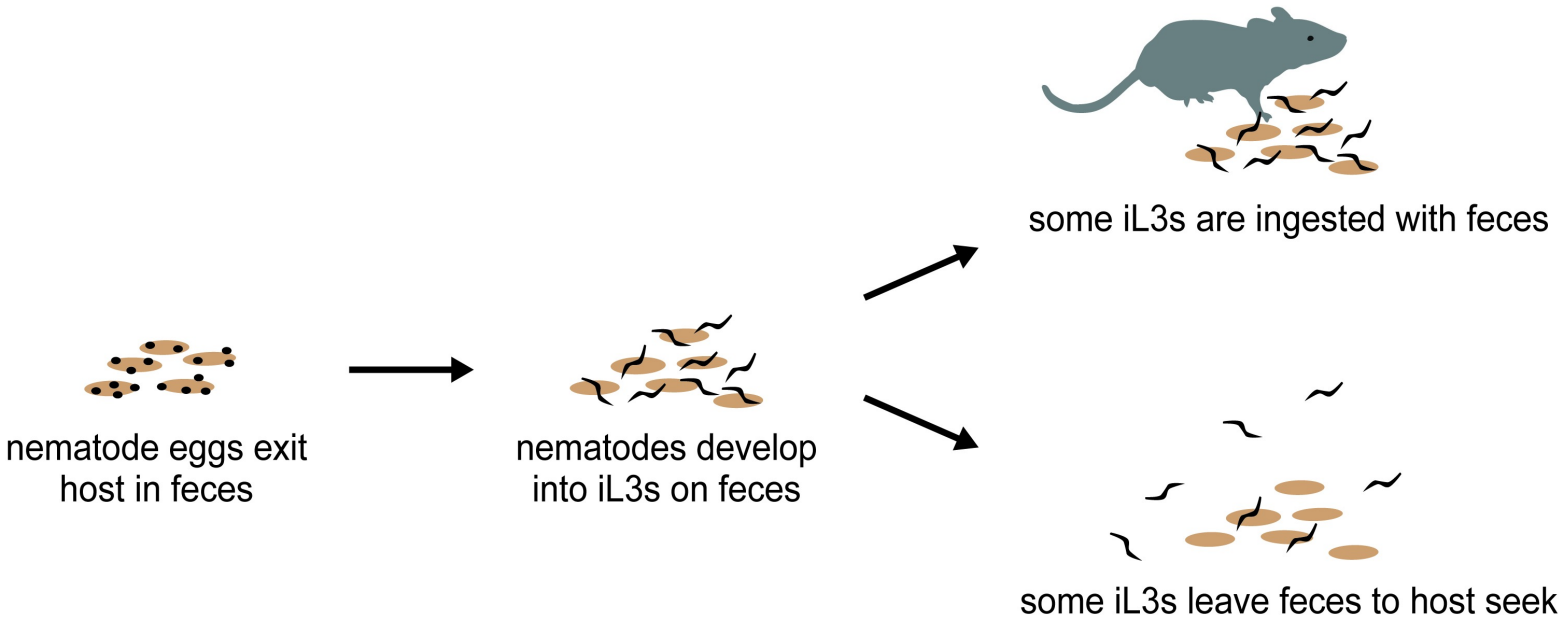
A model for host-seeking behavior in *H*. *polygyrus*. Nematode eggs exit the host in feces, and larvae develop on feces to the iL3 stage. iL3s either infect hosts from the feces on which they developed, or migrate off the feces and into the environment to search for hosts or new host fecal sources. Eventually, nearly all iL3s leave their original fecal source to engage in host seeking.

The robust attraction of *H*. *polygyrus* iL3s to fecal odor could serve to keep some of the iL3s on favorable fecal sources, or to direct them away from suboptimal fecal sources toward more favorable sources. However, we found that even when iL3s are placed on fresh feces, which is presumably a favorable fecal source, approximately half of the population migrates off of the feces within an hour (Fig 2E). Moreover, we found that nearly all iL3s eventually leave their original fecal source to engage in environmental navigation (Fig 2F). These results suggest that all *H*. *polygyrus* iL3s are capable of engaging in environmental navigation, and that if they are not ingested with feces shortly after reaching the iL3 stage, they will leave their original fecal source and disperse into the environment. Once in the environment, they use olfactory cues to migrate toward hosts or new fecal sources (Fig 7).

At the population level, this behavioral flexibility may help to ensure maximal infection rates. Remaining on a known fecal source can in some cases be beneficial: if that fecal source is in or near a nest, the iL3s may encounter hosts by remaining in the nest. However, this behavioral strategy also carries risk: many mice forage and deposit feces far from their nests, in locations where the iL3s are less likely to encounter a mouse using a “sit-and-wait” strategy [34]. Under these circumstances, first dispersing from feces and then using host-emitted sensory cues to migrate toward new hosts or fecal sources is likely to be essential to continue the life cycle. Thus, maximal parasite survival may be achieved when iL3s that do not immediately encounter a host actively disperse in search of hosts. In future studies, it will be interesting to determine whether nematodes that exit the host early in an infection cycle show different dispersal behavior than nematodes that exit the host late in an infection cycle, or whether nematodes that emerge from hosts with a heavier worm burden show different dispersal behavior than nematodes that emerge from hosts with a lighter worm burden.

What is the mechanism that drives some iL3s to migrate off feces, and subsequently toward new hosts or fecal sources? We speculate that olfactory plasticity may function as this mechanism. We have shown that *H*. *polygyrus* iL3s display experience-dependent olfactory plasticity: some odorants are repulsive to iL3s that have been cultured on feces but attractive to iL3s that have been cultured off feces for a week (Fig 5). Repulsion of iL3s from odorants such as CO_2_ and benzaldehyde, which are emitted by host feces [18, 39], may cause the iL3s to migrate off of their original fecal source and disperse into the environment. Once the iL3s have been in the environment for multiple days, these odorants become attractive, likely driving the iL3s toward new hosts or fecal sources.

The shift from repulsion to attraction for both CO_2_ and benzaldehyde response is mediated by environmental CO_2_ levels (Fig 5C and 5E). When iL3s are removed from feces but cultured in the presence of high CO_2_, they remain repelled by both CO_2_ and benzaldehyde. However, when iL3s are removed from feces and cultured at ambient CO_2_, they become attracted to CO_2_ and benzaldehyde. These results suggest that environmental CO_2_ levels may be used as a proxy for the presence or absence of feces.

We found that like *H*. *polygyrus* iL3s, *H*. *contortus* iL3s show experience-dependent modulation of their CO_2_-evoked behavior. *H*. *polygyrus* iL3s showed a shift in their CO_2_ response from repulsive to attractive following removal from feces (Fig 5), while *H*. *contortus* iL3s showed a shift in their CO_2_ response from neutral to attractive (Fig 6A). Thus, in both cases, CO_2_ attraction is likely to be observed in nature in iL3s that have migrated off of feces and are engaging in environmental navigation. In contrast to the passively ingested nematodes tested, the skin-penetrating nematodes tested did not show experience-dependent modulation of their CO_2_-evoked behavior (Fig 6B and 6C). Thus, experience-dependent plasticity based on the presence or absence of feces may be specific to passively ingested nematodes. The differences in CO_2_-evoked behavior between passively ingested iL3s and skin-penetrating iL3s are consistent with their different ecologies. Skin-penetrating iL3s infect primarily via the skin, which emits low levels of CO_2_ [49], so CO_2_ attraction may not be beneficial for skin-penetrating iL3s regardless of their cultivation conditions. Passively ingested nematodes infect via the mouth, which emits high levels of CO_2_ [50]. Thus, in the case of passively ingested nematodes, repulsive or neutral responses to CO_2_ by iL3s on feces may initially drive them off feces, while subsequent attractive responses to CO_2_ may drive them toward the mouths of respiring hosts. *H*. *contortus* is one of the most economically significant livestock parasites worldwide [5], and drug resistance resulting from repeated use of anthelmintic drugs is already a major challenge in combatting infections [9]. Our finding that the olfactory responses of *H*. *contortus* are experience-dependent could facilitate the development of odor-based traps or repellents that could be used in combination with grazing management interventions [51, 52] to prevent nematode infections.

The circuit mechanisms that drive experience-dependent valence changes in passively ingested nematodes remain to be determined. In *C*. *elegans*, CO_2_-response valence is also subject to experience-dependent modulation: adults cultured at ambient CO_2_ are repelled by CO_2_, while adults cultured at high CO_2_ are attracted to CO_2_ [53]. Both CO_2_ attraction and CO_2_ repulsion by *C*. *elegans* are mediated by the BAG sensory neurons in the head and a group of downstream interneurons. The CO_2_-evoked activity of these interneurons is subject to experience-dependent modulation, enabling them to generate opposite behavioral responses to CO_2_ [53]. Since sensory neuroanatomy is generally conserved across nematode species [13], similar circuit mechanisms may operate in passively ingested parasitic nematodes to regulate CO_2_-response valence.

The molecular mechanisms that drive experience-dependent valence changes in passively ingested nematodes are also not yet known. In *C*. *elegans*, CO_2_-response valence is regulated by neuropeptide signaling [53]. However, CO_2_-response valence in *C*. *elegans* changes over the course of hours [53], while CO_2_-response valence in passively ingested parasitic nematodes changes over the course of days (Figs 5B and 6A). Thus, the valence change in parasitic nematodes could involve changes in gene expression and/or neuronal wiring, which occur on a slower timescale than neuropeptide signaling [54–59]. Elucidating the mechanisms that operate in passively ingested nematodes to control olfactory valence will require the development of genetic engineering techniques for these species, which have so far remained intractable to molecular genetic manipulation [60]. Targeted mutagenesis using the CRISPR-Cas9 system has now been achieved in *Strongyloides* species [60–61], and may be applicable to other types of parasitic nematodes in the future.

Entomopathogenic nematodes and skin-penetrating nematodes also show olfactory plasticity, but in response to changes in their prior cultivation temperature [62]. In addition, the entomopathogenic nematode *Steinernema scapterisci* shows age-dependent olfactory plasticity in its response to CO_2_: CO_2_ changes from a repulsive cue in young iL3s to an attractive cue in older iL3s [62]. Thus, olfactory plasticity may be a general feature of parasitic nematode behavior that enables iL3s to modulate their sensory responses based on internal or external conditions so as to increase their chances of encountering a host.

Passively ingested nematodes comprise a group of human and livestock parasites whose behaviors have remained elusive. Increased drug resistance [6–9] necessitates the development of new strategies for their control. Our results suggest that passively ingested nematodes engage in robust and dynamic odor-driven host-seeking behaviors. A better understanding of these behaviors may lead to new strategies for preventing infections.

## Materials and methods

### Ethics statement

*H*. *polygyrus* was passaged in mice, *S*. *stercoralis* was passaged in gerbils, and *A*. *ceylanicum* was passaged in hamsters. All procedures and protocols were approved by the UCLA Office of Animal Research and Oversight (Protocol 2011-060-13B), which adheres to the standards of the AAALAC and the *Guide for the Care and Use of Laboratory Animals*.

### Nematodes and mammalian hosts

*Heligmosomoides polygyrus* (also called *Heligmosomoides bakeri* [27]) was generously provided by Dr. Raffi Aroian (University of Massachusetts Medical School). *Strongyloides stercoralis* (UPD strain) was generously provided by Dr. James Lok (University of Pennsylvania), *Ancylostoma ceylanicum* (Indian strain, US National Parasite Collection Number 102954) was generously provided by Dr. John Hawdon (George Washington University), and *Haemonchus contortus* was generously provided by Dr. Anne Zajac (Virginia-Maryland College of Veterinary Medicine). Male or female C57BL/6 mice for propagation of *H*. *polygyrus* were obtained from the UCLA Division of Laboratory Animal Medicine Breeding Colony. Male Mongolian gerbils for propagation of *S*. *stercoralis* and male Syrian golden hamsters for propagation of *A*. *ceylanicum* were obtained from Envigo. *H*. *contortus* was not propagated in our laboratory.

### Maintenance of *H*. *polygyrus* in mice

*H*. *polygyrus* was serially passaged in C57BL/6 male or female mice as described [30] and maintained on fecal-charcoal plates as described [18]. Briefly, mice were inoculated with ~150 iL3s administered in 100 μL ddH_2_O by oral gavage. Feces infested with *H*. *polygyrus* were collected between days 10 and 60 post-inoculation. Feces were obtained by placing mice overnight on wire cage bottoms above damp cardboard, and collecting the pellets from the cardboard the following morning. Fecal pellets were mixed with dH_2_O and autoclaved charcoal granules to make fecal-charcoal plates. Plates were stored at room temperature until use. iL3s used for behavioral analysis were collected from fecal-charcoal plates using a Baermann apparatus [63]. iL3s cultured “on feces” were collected from fecal-charcoal plates on day 14 (with day 0 being the day of fecal collection) and tested immediately; iL3s cultured “off feces” were collected from fecal-charcoal plates on day 7, incubated in dH_2_O for 7 days at room temperature, and tested on day 14. For the odorant chemotaxis assays in Fig 3, iL3s were either collected from fecal-charcoal plates on days 7–14 and tested immediately or collected on days 7–14 and stored for up to 10 days in dH_2_O at 4°C prior to testing (storage at 4°C in dH_2_O is a standard cultivation condition for *H*. *polygyrus* [30]). In all cases where differences were observed following storage in dH_2_O at 4°C, the data from iL3s stored at 4°C in dH_2_O was excluded from the analysis. For the “off feces” time course in Fig 5, iL3s were collected from fecal-charcoal plates on day 7, incubated in dH_2_O for the indicated number of days, and then tested. For assays involving iL3s cultured at 2.5% CO_2_ either on or off feces, iL3s were collected from fecal-charcoal plates on day 7. iL3s for the on-feces condition were placed onto new fecal-charcoal plates containing autoclaved feces, stored in a CO_2_ incubator with 2.5% CO_2_ for 7 days, and collected from the fecal-charcoal plates using a Baermann apparatus immediately prior to testing. iL3s for the off-feces condition were incubated in dH_2_O in a CO_2_ incubator with 2.5% CO_2_ for the indicated number of days and then tested.

### Culturing of *H*. *contortus*

*H*. *contortus* was maintained on fecal-charcoal plates as described [18]. Plates were stored in an incubator at 23°C until use. iL3s used to test CO_2_ response in Fig 6A were either cultured on fecal-charcoal plates for up to 9 weeks and then tested immediately; or removed from feces, stored in dH_2_O for up to 5 weeks, and then tested. Notably, iL3s maintained on feces and tested immediately showed a neutral response to CO_2_ regardless of their age, demonstrating that the CO_2_ attraction of off-feces iL3s was due to their removal from feces and not their age.

### Maintenance of *S*. *stercoralis* in gerbils

*S*. *stercoralis* was serially passaged in male Mongolian gerbils and maintained on fecal-charcoal plates as described [18]. Briefly, gerbils were inoculated with ~2,250 iL3s in 200 μL sterile PBS by subcutaneous injection. Feces infested with *S*. *stercoralis* were collected between days 14 and 45 post-inoculation. Feces were harvested and mixed with autoclaved charcoal granules to make fecal-charcoal plates as described above. Plates were stored in an incubator at 23°C until use. iL3s used to test CO_2_ response in Fig 6B were cultured on fecal-charcoal plates until day 7; they were then either tested immediately, stored in BU saline [64] for 1 week and then tested, or stored in BU saline for 2 weeks and then tested.

### Maintenance of *A*. *ceylanicum* in hamsters

*A*. *ceylanicum* was serially passaged in male Syrian golden hamsters and maintained on fecal-charcoal plates as described [18]. Briefly, hamsters were inoculated with ~100 iL3s in 100 μL sterile ddH_2_O by oral gavage. Feces infested with *A*. *ceylanicum* were collected between days 14 and 45 post-inoculation. Feces were harvested and mixed with autoclaved charcoal granules to make fecal-charcoal plates as described above. Plates were stored in an incubator at 23°C until use. iL3s used to test CO_2_ response in Fig 6C were cultured on fecal-charcoal plates until day 10; they were then either tested immediately, stored in BU saline [64] for 1 week and then tested, or stored in BU saline for 2 weeks and then tested.

### Short-term dispersal assays for *H*. *polygyrus*

Short-term dispersal assays without feces (Figs 1A and 4A) were performed essentially as described [18]. For each trial, ~50–100 iL3s were placed on a 10-cm chemotaxis plate [65] on a vibration-reducing platform and allowed to disperse for either 1 hour (Fig 1A) or 10 minutes (Fig 4A) in the absence of applied sensory stimuli. The number of iL3s in the outer zone of the plate (the region that excludes a 4-cm-diameter circle at the center of the plate) was then determined. For short-term fecal dispersal assays (Fig 2E), fresh fecal pellets were collected the morning of the assay from uninfected animals. One fecal pellet (~0.03 g) was placed in the center of a 10-cm chemotaxis plate. 15–40 iL3s were pipetted onto the pellet. The plates were then placed on a vibration-reducing platform for 1 hour. The number of iL3s either on the feces, off the feces but within a 4-cm-diameter circle around the feces (zone 1), or outside a 4-cm-diameter circle around the feces (zone 2) was then determined (Fig 2E). iL3s were not visible when they were on the fecal pellet, so the number of iL3s remaining on the feces at the end of the assay was determined by subtracting the number of iL3s in zones 1 and 2 from the total number of iL3s added to the feces. Note that for all dispersal assays, the outermost zone included the walls of the plate, which functioned as a trap such that most of the iL3s that crawled onto the walls of the plate remained there for the duration of the assay.

### Long-term fecal dispersal and nictation assays for *H*. *polygyrus*

Long-term fecal dispersal assays (Fig 2F) were performed by first collecting fresh feces from infected animals; feces were collected as described above, but from a 4-hour collection period. Feces were collected from host animals that were each infected with ~75 iL3s. Individual fecal pellets of similar size were cut in half; one-half of a fecal pellet was then placed on each chemotaxis assay plate and incubated at room temperature. Every 24 hours (within a 3-hour window), the number of animals that had migrated out of the feces and onto the chemotaxis plate was quantified. After quantification, fecal pellets were transferred to fresh chemotaxis plates. On day 10, the fecal pellets were dissociated and the number of iL3s remaining in the fecal pellet was quantified. These numbers were then used to calculate the total number of worms that started out on each fecal pellet, and the cumulative percentage of worms that migrated off the fecal pellet each day. Nictation rates were also determined for each day by counting the number of worms observed to be nictating on the fecal pellet at each time of observation. These numbers were used, in combination with the number of worms remaining on the fecal pellet for each day (calculated as described above), to calculate the percentage of worms nictating on the fecal pellets at each time of observation (S3 Fig).

### Automated tracking of worm movement

Automated tracking was performed as described [18]. For each recording session, 10–15 iL3s were placed on a chemotaxis plate and allowed to acclimate for 10 minutes. iL3 movement was then captured for 20 seconds using an Olympus E-PM1 digital camera attached to a Leica S6 D microscope. WormTracker and WormAnalyzer [35] were used to quantify crawling speed. WormTracker and WormAnalyzer settings were previously described [18].

### Nictation assay

The nictation assays shown in Figs 1C and 4C were performed essentially as described (S2 Fig) [18, 62]. Briefly, agar chips for nictation assays were made from polydimethylsiloxane (PDMS) molds [36]. Chips were approximately 3 cm x 3.5 cm and contained near-microscopic posts that allowed the iL3s to stand. Chips were made using 4% agar dissolved in ddH_2_O. Once the agar had solidified, chips were placed at 37°C for 2 hours followed by room temperature for 1 hour. 10–20 iL3s were transferred to the center of the chip in a 5 μL drop of dH_2_O and allowed to acclimate for 10 minutes. Individual iL3s were then monitored for 2 minutes, and the number of iL3s that nictated during the observation period was recorded. Nictation was defined as an iL3 raising at least half of its body off the plate for at least 5 seconds (S2 Fig).

### Fecal, odorant, and CO_2_ chemotaxis assays

Chemotaxis assays were performed on chemotaxis plates as described [18, 44]. For fecal and odorant chemotaxis assays, 2 μL 5% sodium azide was placed into the center of each scoring region. For fecal chemotaxis assays, feces were obtained from an overnight fecal collection. For assays involving feces from uninfected vs. infected animals (Fig 2D, right), feces were obtained from a 4-hour fecal collection. The feces were then incubated for 3 days at room temperature in a 10-cm Petri dish on filter paper moistened with 1 mL ddH_2_O to prevent desiccation. For assays involving fresh vs. aged feces (Fig 2D, center), feces were obtained from a 4-hour fecal collection and stored in a 10-cm Petri dish without filter paper. “Fresh feces” refers to feces that were used on the day of collection, while “aged feces” refers to feces that were incubated in the Petri dish for 1 day. For all fecal assays, the feces were moistened to a paste with ddH_2_O. 0.5-cm squares of filter paper were affixed to the lid of a chemotaxis plate using double-stick tape. 0.25 g fecal paste was placed onto one of the filter paper squares, and either 50 μL ddH_2_O (for normal fecal chemotaxis assays) or 0.25 g of feces (for fecal competition chemotaxis assays) was added to the other square.

For odorant chemotaxis assays (S4 Fig), 5 μL odorant was pipetted into the center of one scoring region and 5 μL control (paraffin oil, ddH_2_O, or ethanol) was pipetted into the center of the other scoring region. Liquid odorants were tested undiluted. Solid odorants were dissolved to test concentrations as follows: tetradecanoic acid, indole, and 3-methylindole were diluted 0.05 g in 2.5 mL ethanol; octadecanoic acid was diluted 1 g in 80 mL ethanol; L-lactic acid was diluted 0.05 g in 2.5 mL ddH_2_O; and ammonia was purchased as a 2 M solution in ethanol. ddH_2_O was used as a control for L-lactic acid; ethanol was used as a control for tetradecanoic acid, octadecanoic acid, indole, 3-methylindole, and ammonia; and paraffin oil was used as a control for all other odorants. For CO_2_ chemotaxis assays, gases were delivered at a rate of 0.5 mL/min through holes in the plate lids as previously described [18, 44]. Gas stimuli were obtained from Airgas, and consisted of the test concentration of CO_2_, 21% O_2_, and the balance N_2_. Air controls consisted of 21% O_2_ and 79% N_2_. The test concentration of CO_2_ consisted of 15% CO_2_ for *H*. *contortus* and 10% CO_2_ for all other species, unless otherwise indicated.

For all chemotaxis assays, ~200 iL3s were pipetted onto the center of the chemotaxis plate and allowed to distribute in the stimulus gradient on a vibration-reducing platform for 3 h (for fecal and odorant chemotaxis assays) or 1 h (for CO_2_ assays). The number of iL3s in each scoring region was then quantified and a chemotaxis index was calculated as: (# iL3s at stimulus–# iL3s at control) / (# iL3s at stimulus + control). At least two identical assays were always performed simultaneously with the stimulus gradient oriented in opposite directions to control for directional bias due to room vibration or other causes; the pair of assays was discarded if the difference in the chemotaxis indices for the pair of plates was ≥0.9 or if either of the plates had <7 iL3s in the scoring regions. For the odorant chemotaxis assays in Fig 3, significance was calculated relative to a paraffin oil control.

### Data analysis

Statistical analysis was performed using GraphPad Prism or PAST [66]. For each experiment, the D’Agostino-Pearson omnibus normality test was used to determine whether the data were normally distributed. If the data were normally distributed, parametric tests were used; otherwise, non-parametric tests were used. Graphs show medians and interquartile ranges to accurately depict the distribution and variance in our datasets. The heatmap in S5A Fig was generated using Heatmap Builder [67].

## Supporting information

S1 Fig The life cycle of *H*. *polygyrus*. iL3s infect when they are ingested by a mouse, either during fecal consumption or during grooming [30, 34]. The nematodes develop to adulthood in the mouse. Adults reproduce in the intestine, and nematode eggs exit the mouse in feces. The nematodes then develop on feces to the iL3 stage [30]. L1-L4 = 1^st^-4^th^ larval stages. Figure design was adapted from Gang *et al*., 2016 [13].
(PDF)

S2 Fig A nictation assay for *H*. *polygyrus* iL3s. For the nictation assays described in Figs 1C and 4C, iL3s were placed on near-microscopic agar posts [36]. iL3s were allowed to acclimate to the posts for 10 minutes. The number of iL3s that nictated during a 2-minute period was then recorded. Nictation was defined as the iL3 raising at least half of its body off of the plate for at least 5 seconds. Photos show *H*. *polygyrus* iL3s either crawling but not nictating (left), or during different stages of nictation (center and right). Note that the iL3s can crawl between or over the posts, and can stand either on or between the posts. Scale bar = 500 μm. Figure design was adapted from Lee *et al*., 2012 [36].
(PDF)

S3 Fig Nictation of *H*. *polygyrus* iL3s on their original fecal source. Individual fecal half-pellets from infected animals were examined each day over the course of 7 days, and the number of nematodes nictating at each time of observation was determined. Nictation was observed primarily on day 5 post-fecal collection. Nictation frequencies could not be determined beyond day 7 because nearly all of the nematodes had migrated off of the feces by this time (Fig 2F). n = 13 trials.
(PDF)

S4 Fig A chemotaxis assay for iL3s. Stimulus is delivered to one side of the plate and control to the other side (black dots). For odorant chemotaxis assays, the odorant and control were placed directly on the surface of the plate. For CO_2_ chemotaxis assays, CO_2_ and an air control were delivered through holes in the plate lid. iL3s were placed at the center of the plate (double-sided arrow). After 1 hour (for CO_2_-chemotaxis assays) or 3 hours (for odorant-chemotaxis assays), the number of iL3s in each scoring region (circles) was counted, and a chemotaxis index was calculated as indicated. The chemotaxis index ranges from +1 (maximal attraction) to -1 (maximal repulsion).
(PDF)

S5 Fig A comparison of the olfactory preferences of different nematode species. **A**. Olfactory preferences vary across nematode species. Responses are shown as a heat map according to the scale shown at the lower right. Data for *H*. *polygyrus* are from Fig 3; data for all other species are from Castelletto *et al*., 2014 [18]. Odorant order was determined by hierarchical cluster analysis (paired-group algorithm with Euclidean distance as a similarity measure, cophenetic correlation coefficient = 0.71). **B**. Olfactory preferences reflect host range rather than genetic relatedness. The behavioral dendrogram was constructed based on the olfactory behaviors of each species. Hierarchical cluster analysis was performed using a paired-group algorithm with Euclidean distance as a similarity measure, cophenetic correlation coefficient = 0.90. Nematode species are color-coded according to the key shown below the dendrogram. All species being compared have a developmentally arrested third-larval stage that engages in environmental navigation.
(PDF)

S6 Fig CO_2_ response of *H*. *polygyrus* iL3s across concentrations. On-feces iL3s were repelled by CO_2_ (left) and off-feces iL3s were attracted to CO_2_ (right) across concentrations in a CO_2_-chemotaxis assay. *p<0.05, **p<0.01, ***p<0.001, Kruskal-Wallis test with Dunn’s post-test. n = 6–12 trials for each condition. Graphs show medians and interquartile ranges.
(PDF)
