# Supplementary figures and images for: Experience-dependent olfactory behaviors of the parasitic nematode Heligmosomoides polygyrus
Source: PLoS Pathog. 2017 Nov 30;13(11):e1006709. doi: 10.1371/journal.ppat.1006709 (PMC5708605; doi:10.1371/journal.ppat.1006709)

**S1 Fig**

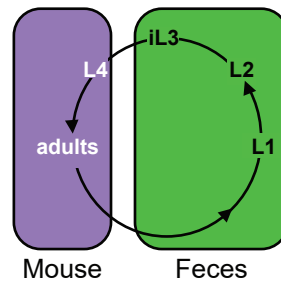

Supplement: S1 Fig — iL3s infect when they are ingested by a mouse, either during fecal consumption or during grooming [30, 34]. The nematodes develop to adulthood in the mouse. Adults reproduce in the intestine, and nematode eggs exit the mouse in feces. The nematodes then develop on feces to the iL3 stage [30]. L1-L4 = 1st-4th larval stages. Figure design was adapted from Gang et al., 2016 [13]. (PDF) [file ppat.1006709.s001.pdf]

S2 Fig

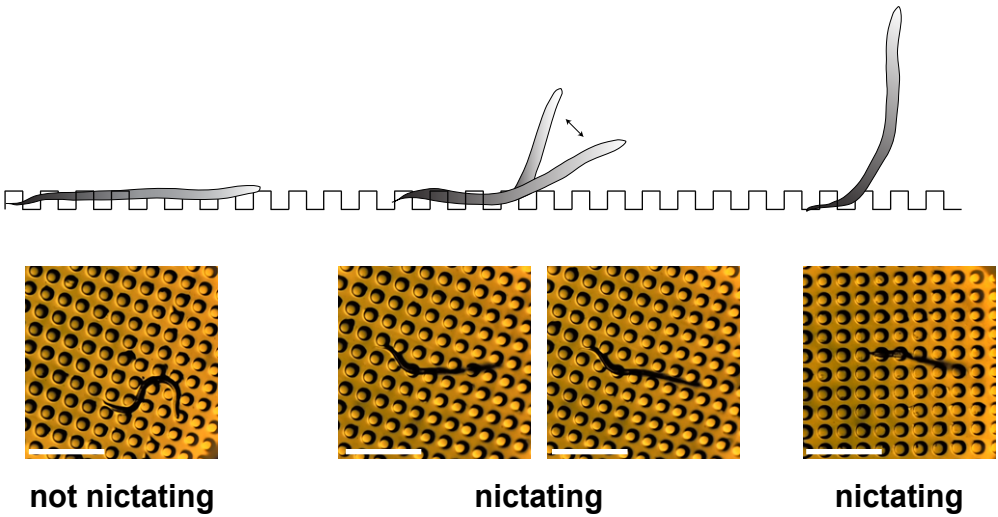

Supplement: S2 Fig — For the nictation assays described in Figs 1C and 4C, iL3s were placed on near-microscopic agar posts [36]. iL3s were allowed to acclimate to the posts for 10 minutes. The number of iL3s that nictated during a 2-minute period was then recorded. Nictation was defined as the iL3 raising at least half of its body off of the plate for at least 5 seconds. Photos show H. polygyrus iL3s either crawling but not nictating (left), or during different stages of nictation (center and right). Note that the iL3s can crawl between or over the posts, and can stand either on or between the posts. Scale bar = 500 μm. Figure design was adapted from Lee et al., 2012 [36]. (PDF) [file ppat.1006709.s002.pdf]

S3 Fig

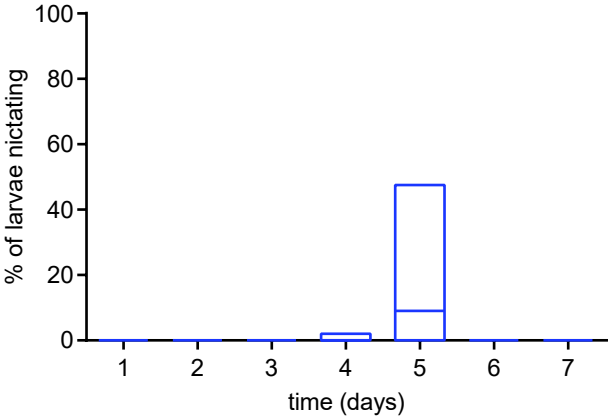

Supplement: S3 Fig — Individual fecal half-pellets from infected animals were examined each day over the course of 7 days, and the number of nematodes nictating at each time of observation was determined. Nictation was observed primarily on day 5 post-fecal collection. Nictation frequencies could not be determined beyond day 7 because nearly all of the nematodes had migrated off of the feces by this time (Fig 2F). n = 13 trials. (PDF) [file ppat.1006709.s003.pdf]

A Olfactory responses across species

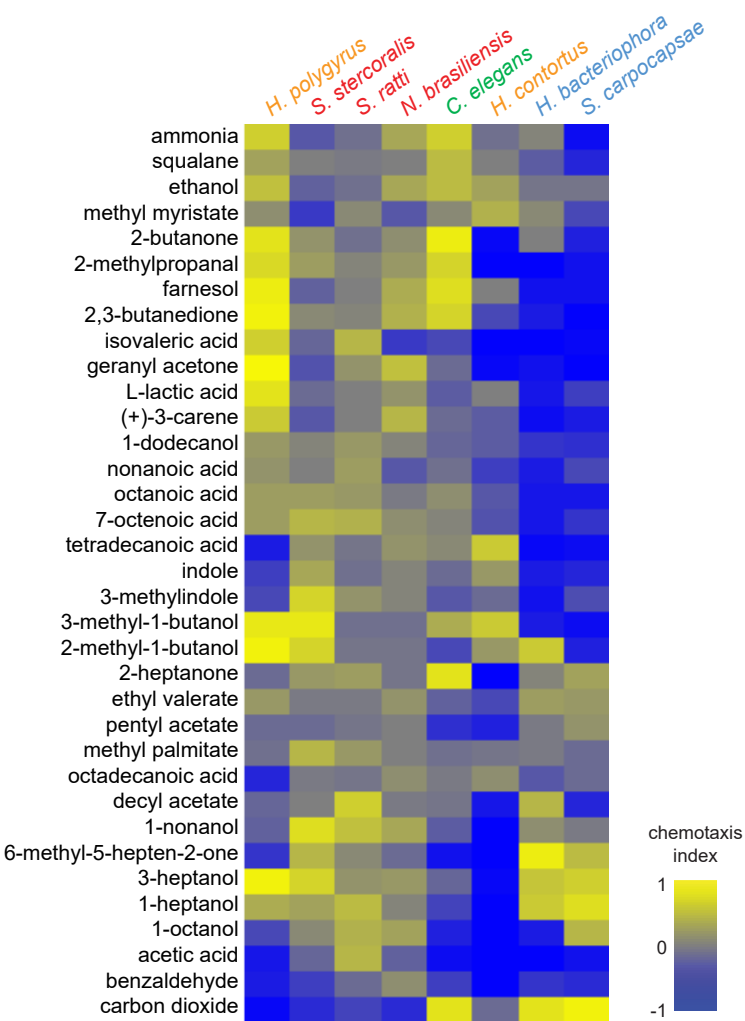

B Similarity of olfactory preferences

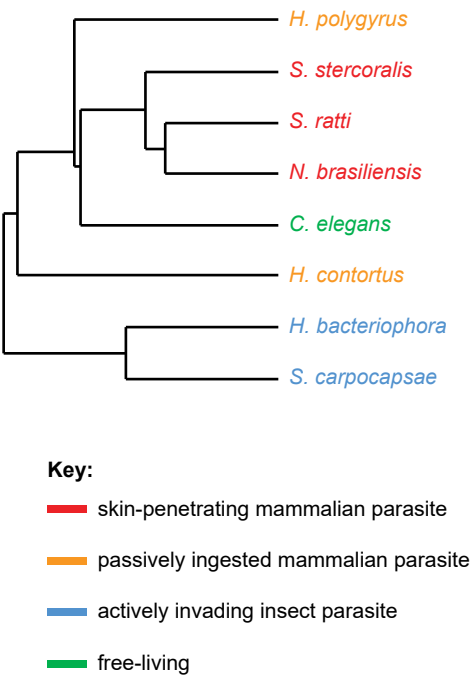

Supplement: S5 Fig — A. Olfactory preferences vary across nematode species. Responses are shown as a heat map according to the scale shown at the lower right. Data for H. polygyrus are from Fig 3; data for all other species are from Castelletto et al., 2014 [18]. Odorant order was determined by hierarchical cluster analysis (paired-group algorithm with Euclidean distance as a similarity measure, cophenetic correlation coefficient = 0.71). B. Olfactory preferences reflect host range rather than genetic relatedness. The behavioral dendrogram was constructed based on the olfactory behaviors of each species. Hierarchical cluster analysis was performed using a paired-group algorithm with Euclidean distance as a similarity measure, cophenetic correlation coefficient = 0.90. Nematode species are color-coded according to the key shown below the dendrogram. All species being compared have a developmentally arrested third-larval stage that engages in environmental navigation. (PDF) [file ppat.1006709.s005.pdf]

S6 Fig

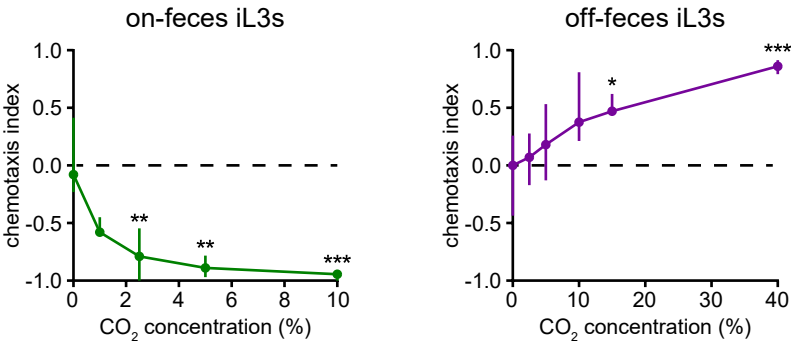

Supplement: S6 Fig — On-feces iL3s were repelled by CO2 (left) and off-feces iL3s were attracted to CO2 (right) across concentrations in a CO2-chemotaxis assay. *p<0.05, **p<0.01, ***p<0.001, Kruskal-Wallis test with Dunn’s post-test. n = 6–12 trials for each condition. Graphs show medians and interquartile ranges. (PDF) [file ppat.1006709.s006.pdf]
