# Supplementary material for: Experience-dependent olfactory behaviors of the parasitic nematode Heligmosomoides polygyrus
Source: PLoS Pathog. 2017 Nov 30;13(11):e1006709. doi: 10.1371/journal.ppat.1006709 (PMC5708605; doi:10.1371/journal.ppat.1006709)
Supplement: S4 Fig — Stimulus is delivered to one side of the plate and control to the other side (black dots). For odorant chemotaxis assays, the odorant and control were placed directly on the surface of the plate. For CO2 chemotaxis assays, CO2 and an air control were delivered through holes in the plate lid. iL3s were placed at the center of the plate (double-sided arrow). After 1 hour (for CO2-chemotaxis assays) or 3 hours (for odorant-chemotaxis assays), the number of iL3s in each scoring region (circles) was counted, and a chemotaxis index was calculated as indicated. The chemotaxis index ranges from +1 (maximal attraction) to -1 (maximal repulsion). (PDF) [file ppat.1006709.s004.pdf]

S4 Fig

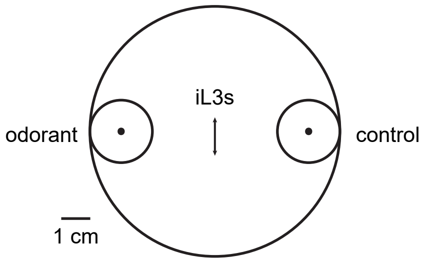

$$\text{chemotaxis index} = \frac{(\#\text{ of iL3s at odorant}) - (\#\text{ of iL3s at control})}{(\#\text{ of iL3s at odorant}) + (\#\text{ of iL3s at control})}$$
